# Effects of preoperative neuromuscular electrical stimulation on quadriceps strength and functional recovery in total knee arthroplasty. A pilot study

**DOI:** 10.1186/1471-2474-11-119

**Published:** 2010-06-14

**Authors:** Raymond J Walls, Gavin McHugh, Donal J O'Gorman, Niall M Moyna, John M O'Byrne

**Affiliations:** 1 Department of Orthopaedic Surgery, Cappagh National Orthopaedic Hospital, Finglas, Dublin, Ireland; 2 Orthopaedic Research Unit, School of Health and Human Performance, Dublin City University, Dublin, Ireland

## Abstract

**Background:**

Supervised preoperative muscle strengthening programmes (prehabilitation) can improve recovery after total joint arthroplasty but are considered resource intensive. Neuromuscular electrical stimulation (NMES) has been shown to improve quadriceps femoris muscle (QFM) strength and clinical function in subjects with knee osteoarthritis (OA) however it has not been previously investigated as a prehabilitation modality.

**Methods:**

This pilot study assessed the compliance of a home-based, NMES prehabilitation programme in patients undergoing total knee arthroplasty (TKA). We evaluated its effect on preoperative and postoperative isometric quadriceps femoris muscle (QFM) strength, QFM cross-sectional area (CSA) and clinical function (subjective and objective). Seventeen subjects were recruited with 14 completing the study (NMES group n = 9; Control group n = 5).

**Results:**

Overall compliance with the programme was excellent (99%). Preoperative QFM strength increased by 28% (p > 0.05) with associated gains in walk, stair-climb and chair-rise times (p < 0.05). Early postoperative strength loss (approximately 50%) was similar in both groups. Only the NMES group demonstrated significant strength (53.3%, p = 0.011) and functional recovery (p < 0.05) from 6 to 12 weeks post-TKA. QFM CSA decreased by 4% in the NMES group compared to a reduction of 12% in the control group (P > 0.05) at 12 weeks postoperatively compared to baseline. There were only limited associations found between objective and subjective functional outcome instruments.

**Conclusions:**

This pilot study has shown that preoperative NMES may improve recovery of quadriceps muscle strength and expedite a return to normal activities in patients undergoing TKA for OA. Recommendations for appropriate outcome instruments in future studies of prehabilitation in TKA have been provided.

## Background

Patients with knee osteoarthritis (OA) have asymmetrical quadriceps femoris muscle (QFM) weakness resulting in significant functional disability. Recent work has shown that muscle activation failure contributes more to quadriceps weakness than muscle atrophy in subjects with advanced knee OA [1]. In turn, greater neuromuscular activation deficits are associated with a reduction in functional capacity [2].

Lower levels of quadriceps femoris muscle (QFM) strength and function prior to total knee arthroplasty (TKA) result in lower functional endpoints after surgery [3]. Further declines in muscle strength following TKA also impact negatively on functional recovery [4-6]. While therapeutic exercise can improve functional capacity in patients with knee OA, research on exercise prehabilitation (preoperative strengthening) in TKA is limited [7-13]. Reported benefits include improved preoperative strength, reduced postoperative length of stay and a greater likelihood of discharge home rather than to a rehabilitation facility [9,12]. In contrast to total hip arthroplasty where significant gains in postoperative functional capacity have been demonstrated [12], only a single case report has cited exercise prehabilitation to improve functional recovery after TKA [13].

Supervised prehabilitation programmes are considered expensive, labour intensive, and pose difficulties for patients regarding transportation and time commitments [9,11,12]. Neuromuscular electrical stimulation (NMES) causes muscle contraction by applying transcutaneous current to terminal branches of the motoneuron [14]. In subjects with knee OA, NMES can increase quadriceps strength and improve functional performance [15], and has been found to be as effective as exercise therapy [16]. As a rehabilitation adjunct post-TKA, NMES can reduce neuromuscular activation deficits and postoperative hospital stay while producing improvements in walking speed and extensor lag [17,18].

Home-based NMES training has not been previously investigated as a prehabilitation modality. It could potentially overcome many of the logistical difficulties associated with supervised exercise programmes. The purpose of this pilot study was to assess the compliance of a NMES prehabilitation programme. Its effects on preoperative and postoperative QFM strength, QFM cross-sectional area (CSA), and clinical function in subjects undergoing TKA will be evaluated.

## Methods

### Patients

Subjects undergoing TKA for end-stage knee OA were recruited from the preoperative assessment clinic of an elective orthopaedic unit between July and October 2007. Exclusion criteria included inflammatory arthritis, morbid obesity (BMI > 40 kg/m^2^), implanted pacemakers or defibrillators, dermatological conditions affecting the thigh, recent participation in an exercise or strength training programme and any neurological disorder or other lower limb impairment affecting function. The ethics review committee of Cappagh National Orthopaedic Hospital gave full approval for this study (ref: RW/03/2007/004) which was performed in compliance with the Helsinki Declaration of 1975, as revised in 2000. All patients provided written informed consent.

Seventeen subjects were recruited (six male, 11 female; mean age, 65.4 years; range, 49 to 80) and randomly assigned to intervention (NMES) or control groups using a computer generated random-number sequence. One patient suffered a postoperative myocardial infarction and another had surgery postponed for management of an unrelated condition. One control subject withdrew from the study due to marked preoperative clinical deterioration. There was no significant difference in baseline demographics for the 14 patients (nine NMES; five control) who completed the study (Table 1).

**Table 1:** Baseline demographics

	Group	
	**NMES**	**Control**

Male : Female	3 : 6	1 : 4

Age (y)	64.4 ± 8.0	63.2 ± 11.4

Height (cm)	161.6 ± 13.9	155.8 ± 5.3

Weight (kg)	80.3 ± 13.6	79.5 ± 14.6

BMI (kg/m^2^)	30.7 ± 3.0	32.8 ± 6.3

Maximum Walking time (mins)	22.8 ± 22.6	17.0 ± 10.4

Values are means ± SD; BMI: Body Mass Index

### Overview

Subjects assigned to the intervention group received 8 weeks of preoperative unsupervised, home-based NMES training applied unilaterally to the QFM of the affected side. Subjects assigned to the control group received standard preoperative care. All subjects received standard postoperative rehabilitation.

QFM strength, objective functional capacity (chair-rise, walk, and stair-climb tests), and subjective outcome measures were assessed at baseline and week 8 preoperatively, and weeks 6 and 12 postoperatively. QFM CSA evaluations were performed at baseline and week 8 preoperatively, and at week 12 postoperatively. One patient in the NMES group did not have preoperative imaging, and was excluded from the CSA analysis.

### Intervention group: NMES

A portable, battery powered, garment-based stimulator (KneeHAB II, Bio-Medical Research, Galway, Ireland) provided the external training stimulus to elicit QFM contraction. It is a duel channel device with a delay of 1 second between their activation at the start and end of the contraction sequence to ensure patellofemoral stability. The control unit, attached directly to the garment, activated the stimulator and modulated stimulation intensity.

The stimulator produced a symmetrical bi-phasic square waveform, with a maximum stimulation intensity of 70 mA and a frequency of 50 pulses.sec^-1^. Pulse width changed dynamically during the stimulation cycle between 100-400 μs. Contraction time (ON) was 5 sec with 10 sec relaxation (OFF) excluding 1 sec ramp-up and 0.5 sec ramp-down. Thus the total cycle length was 16.5 sec. This provided a total ON time of 6.06 min in each 20 min session. The stimulus was provided by a "Multipath" system. This opens pathways between the electrodes for preset periods within each pulse, directing current flow to either medial or lateral electrodes, hence permitting multiple pathways for current flow. It is proposed by the manufacturer that this will allow greater stimulation intensities to be tolerated by a subject and produce greater contraction force while minimising muscle fatigue.

Four reusable self adhering hydrogel electrodes (Axelgaard, Fallbrook, CA) were secured to the inside of the garment with placement over the vastus medialis and vastus lateralis proximally and distally. The surface areas of the four electrodes are: 194 cm^2^; 83 cm^2^; 74 cm^2^; 66 cm^2^.

Subjects assigned to the NMES group received instruction on application of the device to the thigh of the affected limb and usage of the stimulator according to manufacturer guidelines. In addition, they were supplied with clear written instruction on the device controls and the NMES training program schedule. All NMES training sessions were performed in the sitting position with the knee flexed to 60 degrees, the foot flat on the floor, and toes against a wall to permit isometric muscle contraction.

The stimulator was used for 20 min. day^-1 ^on alternate days during the initial two week conditioning period. Patients were encouraged to increase the stimulation intensity to their maximum tolerated level (pain threshold) during each session. The subsequent training period involved a single 20 min session. day^-1^, 5 days. wk^-1 ^for 6 weeks preoperatively with patients encouraged to train at their highest tolerated stimulation intensity. A logbook was provided to all subjects to record usage. In addition and unknown to the patients the stimulator also recorded usage.

### Control group

Subjects assigned to the control group received individual instruction on knee range of motion and quadriceps muscle strengthening exercises from a physiotherapist. Static quadriceps strengthening exercises were performed while lying supine with subjects instructed to pull back their toes, push their knee downwards into the bed and hold for 5 seconds. A similar exercise was demonstrated with a soft cushion roll placed under the knee in an attempt to achieve terminal knee extension and strengthen the inner range quadriceps muscle (vastus medialis [VMO]). In addition, subjects were instructed to perform straight leg raises. Sets of 10-20 repetitions of each exercise performed twice daily were recommended. Instruction was also provided on knee flexion and extension exercises performed both sitting and, if tolerated, while standing. Adherence was not recorded, thereby reflecting normal care.

### Quadriceps muscle strength measurement

A Biodex dynamometer (Biodex Medical Instruments, Shirley, NY) was used to determine QFM maximum voluntary isometric contraction (MVIC) peak torque (Nm) of both limbs [19-21]. The testing position was standardised with a hip angle of 110° and the knee flexed at 60°. The fulcrum of the dynamometer lever arm was aligned with the inferior aspect of the lateral femoral condyle. Waist, thigh, and shoulder straps stabilised patients during testing to minimise additional muscle group substitution. Following three submaximal contractions, subjects performed a set of three consecutive maximal contractions. Each contraction was 5 sec in duration interspaced with a 50 sec rest period. The greatest force generated was recorded as the MVIC. Verbal encouragement ("kick out as hard as you can") was provided by a research assistant blinded to subject group assignment.

### Quadriceps femoris cross-sectional area

A Gyroscan Intera 1.5 T MRI scanner (Philips Medical Systems, Holland) was used to determined QFM CSA of both thighs using a 4 mm slice thickness: 0.4 mm slice gap, 100 ms echo time; and 3000 ms relaxation time. A coronal scouting scan established the level of the mid-thigh using the greater trochanter and lateral knee joint line as anatomical markers. Twelve T2-weighted axial images were produced with a field of view of 30 cm (256 × 256 pixel matrix). Following spatial calibration, a single clinician, blinded to group assignment, used manual planimetry to outline the QFM as the region of interest, with CSA (cm^2^) automatically calculated. The average area of the central two images was recorded as the QFM CSA.

### Objective functional capacity

A straight back chair with adjustable leg height was used for the timed chair-rise test. Patients sat with their knees flexed at 90° and their arms folded across their chest. The time taken to complete three full cycles (stand-sit) was recorded. The 25-metre timed walk test was performed in an indoor hallway with patients allowed to stop or use a mobility aid if required. The stair-climb test was performed on an indoor stairwell with 11 steps (18 cm rise, 30 cm depth). Patients were asked to try and refrain from using the handrails during the test, and the time taken to ascend, turn, and descend was recorded. Each test was performed three times and the fastest time was recorded. All assessments were performed in the same order for each participant with the assessor blinded to group assignment.

### Self-report outcome measures

Individual perception of disability was assessed using the Western Ontario McMaster Osteoarthritis Index (WOMAC) [22]. The WOMAC is a validated health questionnaire specific for hip and knee OA, consisting of 24 questions scored from zero to four (best to worst respectively). These are sub-categorized into pain (0-20), stiffness (0-8), and function (0-68). The Medical Outcome Study short form 36 (SF-36) evaluates general health using a 36 item questionnaire. Component scores are given for physical and mental health, both scored from zero to 100 with a higher score indicating better health. Reliability and validity of the SF-36 are well established [23,24].

### Surgical technique and postoperative management

All TKA's were performed by the senior author (JO'B) under tourniquet control. A midline incision was made with a standard medial parapatellar approach to the joint. A quadriceps snip was not performed in any knee. The PCL was sacrificed in all cases with insertion of the Scorpio PS knee system (Stryker, Limerick, Ireland). All operations were performed under spinal anaesthesia and patients received PCA or epidural analgesia in the immediate postoperative period. All knees were wrapped in a wool and crepe dressing for the first 48 hours postoperatively.

A standardised rehabilitation programme was commenced on the first postoperative day. These involved static quadriceps strengthening exercises, as well as foot and ankle range of motion exercises to help prevent thromboembolic events. On day two postoperatively, the wool and crepe dressing was removed which permitted the addition of knee range of motion exercises. Weight-bearing with the use of a walking frame was also commenced on day two postoperatively. From day three postoperatively until discharge, subjects attended the hospital physiotherapy department twice daily for further individual supervised rehabilitation. If tolerated, straight leg raising exercises were commenced. Length of postoperative hospital admission and discharge location (home or rehabilitation facility) were recorded.

### Statistical analysis

Independent t-tests were used to evaluate potential group differences for age, height, weight, and maximum walking time. The non-parametric Friedman test was used to compare within group differences in strength, CSA, function, clinical evaluations, and subjective outcomes. Post hoc analyses were performed using the Wilcoxon signed ranks test with Bonferroni adjustment for multiple comparisons. Between group comparisons were performed using the Mann Whitney test. The relation between selected parameters was determined using Spearman rho correlations. Statistical significance was set at p = 0.05. Statistical tests were performed using the Statistical Package for Social Sciences version 15.0 (SPSS, Inc., Chicago, IL).

## Results

### Compliance

Patient reported compliance was 99.4% (range, 97.1-100%) and stimulator recorded compliance was 99.0% (range, 77.2-114.8%). Stimulator recorded compliance was more than 97% in all but one male subject.

### Quadriceps femoris muscle strength and CSA

**Figure 1:**
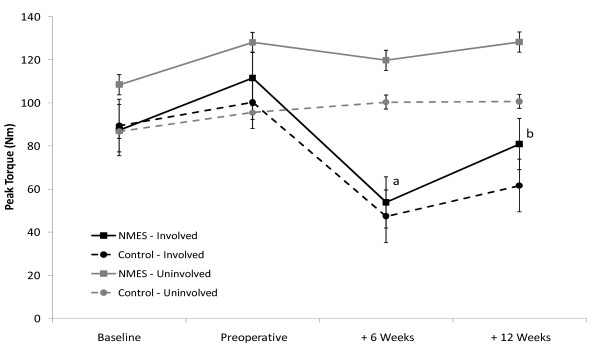
**Quadriceps Femoris Muscle Strength**. ^a^p < 0.05 vs. Preoperative; ^b^p < 0.05 vs. + 6 Weeks; Error Bars represent Standard Error

Preoperatively, QFM peak torque of the involved limb in the NMES group increased by 27.8% (Figure 1); this was close to statistical significance (p = 0.021; alpha level = 0.013). An increase of only 12.1% (p = 0.08) was seen in the control group. QFM strength of the involved limb decreased by approximately 50% in both groups at 6 weeks post-TKA compared to preoperative levels. Only the NMES group experienced significant strength recovery from week 6 to week 12 post-TKA group (53.3%, p < 0.05). There was no change in strength or CSA of the uninvolved QFM in either group over the study period.

**Figure 2:**
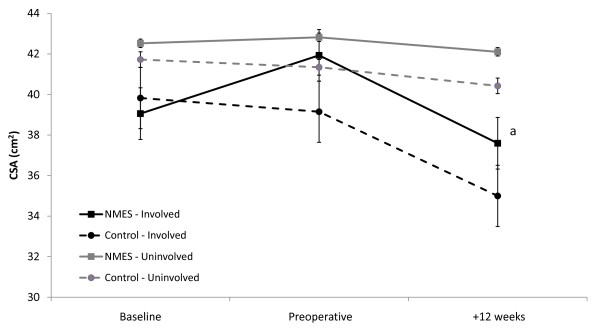
**Quadriceps Femoris Muscle Cross-sectional Area**. ^a^p < 0.05 vs. Preoperative; Error Bars represent Standard Error

Preoperatively, there was a trend towards an increase in QFM CSA of the involved limb in the NMES group (7.4%, p = 0.036; alpha level = 0.017) (Figure 2). Although involved QFM CSA decreased by approximately 10% postoperatively in both groups, it was only significant in the NMES group (p < 0.05). When QFM CSA at 12 weeks post-TKA was compared to baseline levels, it had decreased by 3.7% in the NMES group while a reduction of 12.1% (p > 0.05) was found in the control group. There was no difference in QFM CSA between the treatment groups or between involved and uninvolved limbs in either group over the study period.

### Objective functional capacity

**Figure 3:**
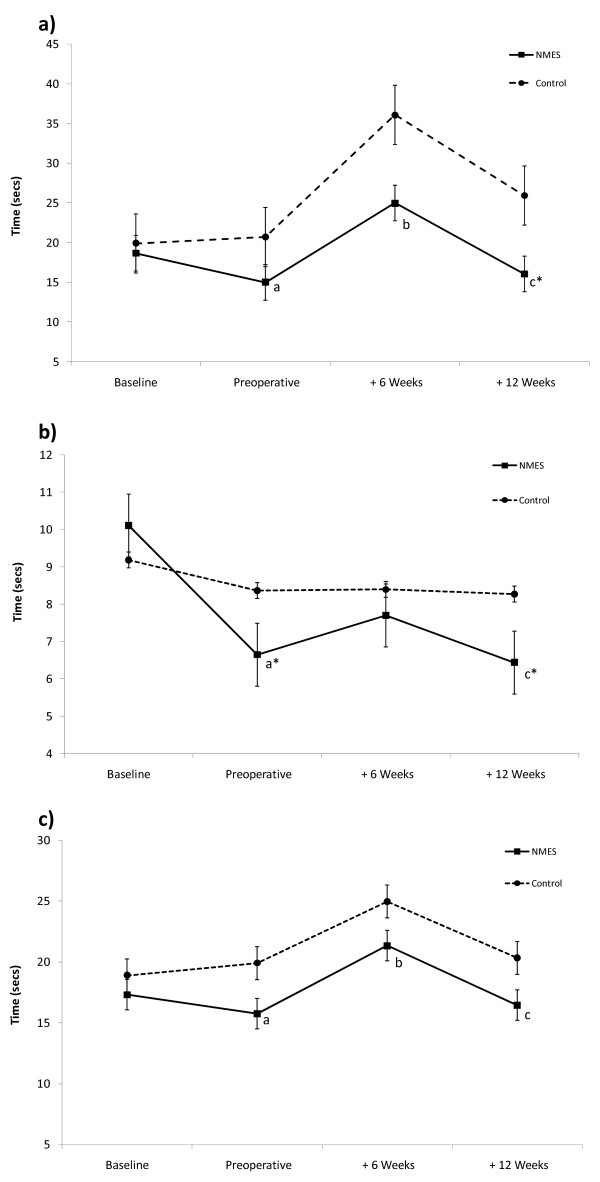
**Objective Functional Capacity**. ^a^p < 0.05 vs. Baseline; ^b^p < 0.05 vs. Preoperative; ^c^p < 0.05 vs. + 6 Weeks; *p < 0.05 vs. Control; Error Bars represent Standard Error **a) Timed Stair-Climb Test. b) Timed Chair-Rise Test. c) 25 metre Timed Walk Test.**

Preoperative improvement in objective functional capacity was only found in the NMES group: timed walk test, 9% (p < 0.05); timed stair-climb test, 19.7% (p < 0.05); timed chair-rise test, 34.2% (p < 0.05) (Figure 3). Performance in the chair-rise test was 25.9% better (p < 0.05) in the NMES than the control group preoperatively. The time required to complete the stair-climb test and walk test increased by 66.8% (p < 0.05) and 35.5% (p < 0.05) respectively in the NMES group at 6 weeks postoperatively compared to preoperative values. Only the NMES group demonstrated significant postoperative functional recovery from week 6 to week 12 postoperatively (walk test: 22.9% [p < 0.05]; stair-climb test: 36.8% [p < 0.05]; chair-rise test: 16.4% [p < 0.05]). Performance was better in the NMES group compared to the control group in the stair-climb test (61.6%, p < 0.05) and chair-rise test (34.2%, p < 0.05) at 12 weeks post-TKA.

### Self-report outcome measures

Postoperatively, SF-36-physical health (p < 0.05) and mental health (p < 0.05) scores improved in the NMES group at week 12 compared to week 6 (Table 2). Improvements were also seen in the NMES group with the SF-36-mental health (p < 0.05) and the WOMAC pain score (p < 0.05) at week 12 post-TKA compared to preoperative levels. All outcome scores of the control group, and the WOMAC function and stiffness subscales of the NMES group did not change significantly over the study period. There were no between-group differences in any of the subjective outcome measures at any time-point.

**Table 2:** Self-report outcome measures

	Time			
	**Baseline**	**Preoperative**	**+ 6 Weeks**	**+ 12 Weeks**

**WOMAC Pain**				
NMES	11.7 ± 2.7	11.4 ± 3.3	7.9 ± 5.9	5.4 ± 4.0 ^a^
Control	10.0 ± 5.7	11.8 ± 4.0	5.4 ± 3.0	5.2 ± 4.3

**WOMAC Function**				
NMES	36.9 ± 11.3	34.7 ± 11.0	29.8 ± 16.9	19.9 ± 13.9
Control	37.0 ± 15.7	38.4 +/- 14.4	24.2 ± 9.9	21.6 ± 11.3

**WOMAC Stiffness**				
NMES	5.0 ± 2.9	5.1 ± 1.8	4.6 ± 2.4	3.2 ± 2.0
Control	4.8 ± 1.6	5.4 ± 1.3	4.6 ± 1.1	3.0 ± 1.4

**SF-36 Physical Health**				
NMES	41.0 ± 22.1	44.4 ± 18.7	35.7 ± 17.0	57.1 ± 18.0 ^b^
Control	39.0 ± 20.8	45.6 ± 17.8	39.0 ± 9.4	57.0 ± 25.2

**SF-36 Mental Health**				
NMES	63.7 ± 18.3	62.3 ± 17.5	58.6 ± 26.0	77.7 ± 14.7 ^b a^
Control	56.4 ± 22.6	68.6 ± 23.1	58.8 ± 19.5	71.0 ± 26.4

Values are means ± SD;

WOMAC - Western Ontario McMaster Osteoarthritis Index; SF-36-Short Form 36

^a^p < 0.05 vs. Preoperative; ^b^p < 0.05 vs. + 6 Weeks

### Postoperative rehabilitation

There was no difference in length of postoperative hospitalisation between the two groups (8.1 days [NMES] versus 8.8 days [control], p = 0.94). Two patients in the NMES group and one patient in the control group were discharged to a rehabilitation facility.

### Correlations

Assessing the cohort collectively, QFM strength was inversely related to the stair-climb test at all time points and to the walk test at baseline, and weeks 6 and 12 postoperatively (Table 3). The association between QFM strength and the chair-rise test was only significant preoperatively (r^2 ^= 0.26; p < 0.05). There were limited associations found between the objective measures of functional capacity and the self-report outcome measures.

**Table 3:** Significant correlations

Comparison	p value	r value
**Quads Strength and TWT**		
Baseline	0.003	-0.688
Week 6	0.003	-0.697
Week 12	0.005	-0.662

**Quads Strength and TST**		
Baseline	0.008	-0.626
Preoperative	0.020	-0.552
Week 6	0.009	-0.618
Week 12	0.001	-0.776

**Quads Strength and TCT**		
Preoperative	0.032	-0.508

**WOMAC-Pain and TCT**		
Baseline	0.047	0.464

**WOMAC-Function and TWT**		
Baseline	0.034	0.252

**SF36-Physical Health and TWT**		
Baseline	0.009	-0.623
Week 12	0.041	-0.481

**SF36-Physical Health and TST**		
Preoperative	0.039	-0.487

**SF36-Mental Health and TCT**		
Week 12	0.026	-0.528

p value - significance level

r value - correlation coefficient

WOMAC - Western Ontario McMaster Osteoarthritis Index

SF-36 - Short Form 36

CSA = Cross-sectional area

TWT = 25-metre timed walk test/TST = Timed stair-climb test/TCT = Timed chair-rise test

## Discussion

This is the first study to assess the use of NMES as a prehabilitation modality. We have demonstrated that adherence to an 8 week preoperative NMES programme in patients undergoing TKA is excellent. The intervention group had a trend towards an increase in quadriceps muscle strength with significant improvements in functional performance following NMES prehabilitation. Postoperatively, these effects translated into earlier strength and functional recovery from week 6 to week 12 in the NMES group. Many observations from this pilot study should be considered when planning future clinical trials of prehabilitation in orthopaedic practice.

Compliance with the NMES programme in this pilot study is superior to previous work [15] that applied NMES to subjects with knee OA (99% vs. 81%). The device we used was garment-based, potentially making it more amenable to training. Some subjects had a stimulator recorded compliance of more than 100%. Given that the stimulator can only function when in contact with skin, participants may have spent additional time familiarising themselves with the device early in the study programme. It is also possible, however, that they trained for more than was prescribed in the study protocol.

The NMES group increased preoperative QFM strength by 28% (p > 0.05) which compares favourably with exercise prehabilitation [9,11,12]. Using a similar home-based NMES programme in patients disabled by knee OA, Talbot et al [15] produced a 9% increase in QFM strength with stimulation intensity gradually increased at predetermined intervals over their training period. The greater strength gains found in the present study may be attributed to a greater training stimulus given that our subjects were encouraged to use the stimulator at their maximal tolerated level throughout the training period. Conversely, the subjects in our study all had end-stage knee OA and may have been generally weaker and potentially more responsive to NMES rehabilitation.

The preoperative improvements in quadriceps muscle strength and objective functional capacity were greater in the NMES group than the control group, although we recognise that a between group effect was only seen with the chair-rise test. While further study is needed, we believe this preliminary data suggests that NMES may confer a greater preoperative benefit than simply providing instruction to patients on preoperative strengthening and range of motion exercises. The beneficial training effect of NMES is further supported by the observed trend towards an increase in preoperative QFM CSA (7.4%).

Previous trials cited QFM strength decreases of around 60% post-TKA [5,6]. In this prospective clinical study, QFM strength of the involved limb decreased in both groups by approximately 50% (P > 0.05) at 6 weeks postoperatively compared to preoperative levels. Hence, NMES prehabilitation may not attenuate early postoperative declines in quadriceps muscle strength. A corresponding early postoperative deterioration in objective functional capacity was only seen in the NMES group and illustrates the early detrimental effects of TKA on functional performance. We are unsure as to why no notable change in functional capacity was observed in the control group over the same postoperative period. It may be that, since they had not experienced an improvement in function preoperatively, there was little capacity for further deterioration in the control group postoperatively.

Significant strength and functional recovery from 6 to 12 weeks postoperatively was only seen in the NMES group. By the end of the study period, the NMES group was 24% stronger compared with the control group and performed better at the stair-climb and chair-rise tests (p < 0.05). Buchner et al [25] described a curvilinear relationship where small changes in muscle strength were associated with substantial improvements in function in frail elderly adults. Extrapolating this to our weakened cohort could explain how significant group differences in functional capacity were found despite group differences in QFM strength being statistically non-significant.

While there is agreement that exercise prehabilitation enhances postoperative strength recovery after TKA, improvements in functional capacity have only been previously described in a case report [11-13]. Differences in assessment methodology could explain this. For example, Rodgers et al [11] used only a short 10-metre timed walk test to assess functional capacity, while Rooks et al [12] employed a timed up and go test which incorporates a single chair-rise with a 6-metre walk. The assessment distance used in these tests may be insufficient to determine a group effect in subjects with advanced knee OA, rendering the methods insensitive. In contrast, we found QFM strength to correlate strongly with our 25-metre walk test as well as the stair-climb. We recommend that both should be performed in future studies that wish to evaluate the efficacy of prehabilitation in TKA.

As in this study, where NMES had a beneficial effect on muscle mass, Rodgers et al [11] saw a reduction in postoperative muscle atrophy with exercise prehabilitation. It would therefore appear that preoperative increases in QFM CSA due to both exercise and NMES prehabilitation may attenuate muscle atrophy following TKA.

We found no significant improvement in the perception of knee pain in subjects with osteoarthritic knees following NMES training. This is consistent with Talbot et al [15], who also did not find NMES to decrease subjective knee pain. Although Durmus et al [16] reported an improvement in knee pain in subjects with knee OA, they excluded those with end-stage disease. Subjects with advanced knee OA may have progressed to a stage where increases in QFM strength cannot improve knee pain. Alternatively, there may simply have been insufficient statistical power in this pilot study to determine a difference.

The data on postoperative knee pain must be interpreted with caution given the sample size limitation. Although we found the NMES group to have a significant reduction in knee pain at 12 weeks compared to preoperative levels there was no between group effect. Previous studies of exercise prehabilitation have also reported no difference in knee pain between control and experimental groups even up to 12 months postoperatively [9,10,12]. Hence, the analgesic effect conferred from the TKA itself is possibly the principal factor in the reduction of knee pain post-operatively with prehabilitation providing little additional benefit.

Changes in subjective functional outcome scores did not reflect changes seen with the objective functional assessments. For example, an improvement was found in the SF-36-physical health scores of the control group preoperatively despite them deteriorating in two objective measures of functional capacity. This is further illustrated by the limited associations found between objective and subjective data. A moderate association between self-report and objective performance measures has been previously described by Maly et al [26] in subjects with knee OA. They concluded that self-report measures relate to knee pain whereas physical performance measures relate to self-efficacy. If we accept that TKA provides a much greater effect in reducing postoperative pain than prehabilitation, then subjective questionnaires would be less sensitive in evaluating prehabilitation. This would explain why previous studies [9,10] that evaluated functional outcomes using subjective measures alone determined no benefit from exercise prehabilitation despite subjects increasing muscle strength preoperatively. Had objective measures of functional capacity been incorporated into these studies, a beneficial effect of prehabilitation may have been found.

Previous work has reported that exercise prehabilitation can shorten the length of hospital stay and increase the possibility of going home on discharge rather than to a rehabilitation facility [9,12]. Based on our data, NMES prehabilitation in the setting of TKA does not reduce immediate postoperative hospitalisation. We recommend further study should be performed on a larger cohort to verify this.

This pilot study employed a randomised control design and all evaluations were performed with assessors blinded to group assignment. Unlike previous studies, we employed several objective measures of function to determine improvement in postoperative recovery, as well as isometric and radiological muscle evaluation. The principle limitation is our small sample size. We recognise that very strict inclusion criteria were employed in this study. NMES could certainly be applied to a wider population in accordance with the manufacturers' guidelines. We have demonstrated that NMES is well tolerated by patients as part of an unsupervised, home-based programme. However, since the sample size is limited with many essential results based on statistical trends, a formal randomised controlled trial is now required to determine if the pre- and postoperative gains in strength and objective functional capacity observed in this clinical trial are reproducible in a larger population.

## Conclusions

In this pilot clinical study of NMES prehabilitation, we found NMES to be well tolerated by patients with advanced knee OA. When used for 8 weeks preoperatively, it appears to increase muscle strength and hasten functional recovery following TKA and also may reduce the extent of postoperative muscle atrophy. The self-report questionnaires did not reflect changes seen with objective functional assessments. They may be less sensitive instruments for evaluating prehabilitation in TKA. Given that no previous data exists on the use of NMES prehabilitation, the results from this pilot study warrant further research on a larger TKA population as part of a cost-analysis and efficacy study. We believe this study provides preliminary evidence that preoperative NMES may expedite a return to normal activities in patients undergoing TKA for knee OA.

## Competing interests

They authors declare that they have no competing interests.

## Authors' contributions

RW was the principle researcher involved in study conception and design as well as implementation, data analysis and interpretation, and manuscript preparation. GM was involved in the study conception and design and the drafting of the manuscript. DO was involved in the study conception and design and the drafting of the manuscript. NM was involved in the study conception, design and implementation as well as data anlysis and interpretation and the drafting of the manuscript. JO was involved in the study conception and design and the revision of the manuscript. All authors read and approved the final manuscript.

## Authors Information

Raymond J Walls, MD, MRCSI, MFSEM, Orthopaedic Specialist Registrar

Gavin McHugh, MB, MRCSI, Orthopaedic Specialist Registrar

Donal J O'Gorman, MSc, PhD, Lecturer in Exercise Physiology

Niall M Moyna, MSc, PhD, FACSM, Professor of Exercise Physiology

John M O'Byrne, MCh, FRCSI, FRCS(Tr. & Ortho.), FFSEM, Professor of Orthopaedic Surgery

## Pre-publication history

The pre-publication history for this paper can be accessed here:

http://www.biomedcentral.com/1471-2474/11/119/prepub
